# Observation of Wavelength Tuning and Bound States in Fiber Lasers

**DOI:** 10.1038/s41598-018-24435-7

**Published:** 2018-04-16

**Authors:** Yang Xiang, Yiyang Luo, Bowen Liu, Zhijun Yan, Qizhen Sun, Deming Liu

**Affiliations:** School of Optical and Electronic Information, National Engineering Laboratory for Next Generation Internet Access System, Huazhong University of Science and Technology, Wuhan, 430074 Hubei P. R. China

## Abstract

We report an experimental observation of wavelength tuning and bound states in fiber lasers. A Mach-Zehnder interferometer (MZI) is adopted as an intra-cavity tunable filter to realize large-scale wavelength tuning and bandwidth controlling. By finely manipulating the MZI and intra-cavity polarization state, continuous wavelength-tunable operation from 1550.7 nm to 1580.8 nm is achieved. Meanwhile, the spectral bandwidth varying from 1.85 nm to 3.41 nm is also controlled by broadening the free spectrum range (FSR) of the MZI. Additionally, with modest polarization adjustment, both tightly and loosely bound states are experimentally observed, which can be validated by the numerical simulations. The results indicate that the proposed fiber laser is attractive for telecommunication systems, on account that the tuning feature can be applied to wavelength-division multiplexer (WDM) and the various soliton bound states could contribute to the high-level modulation format.

## Introduction

Due to the advantages of high repetition rate or pulse energy^1,2^, the dissipative solitons (DSs) mode-locked fiber lasers have been widely studied for improving ultrashort pulsed light sources. Wavelength-tunable mode-locked fiber lasers are interpreted as a desirable candidate for the practical applications such as optical instrumentation, telecommunication systems, all-optical sampling and fiber sensing. In particular, some approaches have been proposed and demonstrated to achieve the wavelength-tuning of mode-locked fiber lasers, such as exploiting cascaded fiber grating cavities^3,4^ or various fixed comb filter structures^5–8^. However, these methods have limitations in continuous wavelength tuning due to their inflexible structures and have severe restriction of wavelength selectivity in practical applications. Then, the modified tunable fiber lasers are studied based on fiber birefringence induced invisible filter^9–15^ or space optical coupling structure^16^. In these ways, more flexible wavelength tuning and wider adjustable range around 20 nm are achieved. Nevertheless, due to the rough change of the laser operation parameter (birefringence), the output spectrum is fluctuating along with the wavelength changing. Meanwhile, the commercial tunable filter as well as free-space-to-fiber coupling is either costly or complicated. Recently, Mach-Zehnder interferometer (MZI) has been applied in fiber laser for generating high repetition rate pulses based on the filter-driven four-wave mixing effect^17^ or providing flexibly tunable filtering function^18^, whose flexible wavelength control also paves another way to implement the wavelength-tunable operation of fiber lasers and match the WDM technique. Besides, the stable structure and wide filtering range can help conducting the useful tunable fiber laser in practical applications.

For the soliton quantization effect, conventional DSs tend to split and form various solitons complexes as the pump power increases, instead of sequentially amplified. Particularly, bound states of solitons (i.e. soliton molecules), characterized by complex waveforms and solitons-bound structure, have been a major research topic in fiber lasers. The origin of soliton molecules can be ascribed to the balance of attractive and repulsive forces between solitons in the mode-locked fiber laser. Since the soliton molecule firstly theoretically studied based on the complex Ginzburg-Landau equation (CGLE) by Malomed^19^, the characteristics of bound-state solitons in various fiber lasers have been widely reported. In negative dispersion regime, the bound states are experimentally observed by Yun^20,21^. Moreover, Zhao *et al*.^22^ expand the observation of bound states to zero-dispersion regime and various bound states of dispersion-managed solitons are systematically recorded. Instead of Nonlinear Polarization Rotation (NPR) technique, the semiconductor saturable absorber mirror (SESAM)^23^ as well as new materials like black phosphorus^24^ is also used to achieve bound states in normal dispersion. In applications, stable bound states can support the versatile applications including realizing “coding and transmission of information in high-level modulation formats” and providing a way to break through the binary coding limits.

In this paper, a wavelength-tunable passively mode-locked fiber laser is developed, incorporating a MZI as the intra-cavity filter. Continuous wavelength-tuning can be achieved through finely adjusting the MZI; meanwhile, the spectral bandwidth is controllable by changing the free spectrum range (FSR) of MZI. Additionally, with modest polarization manipulation, tightly and loosely bound states of solitons are experimentally observed. Further, numerical simulations are conducted to confirm the specific parameters of the soliton molecules including phase difference and pulse separations.

## Results

### Optical filtering characteristics of MZI

**Figure 1:**
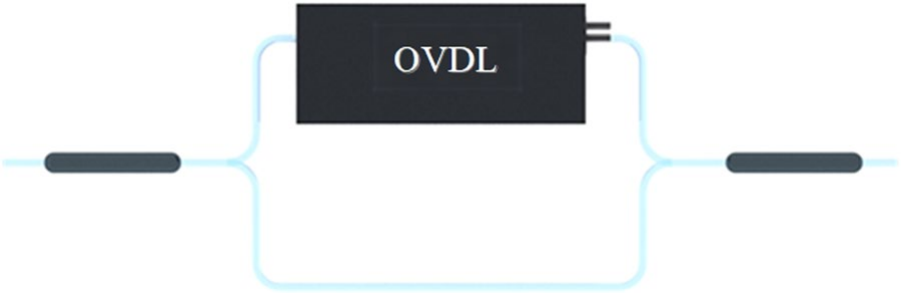
Schematic diagram of the MZI. 3 dB OC, 50:50 optical coupler; OVDL, optical variable delay line.

Schematic diagram of the MZI is shown in Fig. 1 where a commercial optical variable line (OVDL, General Photonics, VDL-001) and a length of standard single-mode fiber are adopted to connect two 3-dB optical couplers. In the structure, two arms of light will go through different optical path along with the changing of the OVDL. When the lights meet in the latter optical coupler, the controllable phase difference can result in multiple interference spectra.

The FSR and 3-dB bandwidth of MZI interference spectrum can be flexibly adjusted. The FSR, based on the theory of wave superposition, can be expressed as:

1 $${\rm{FSR}}=\frac{{\lambda }^{2}}{{\boldsymbol{\Delta }}{\rm{L}}}$$

where $\lambda$ is approximately equal to the operating wavelength; ${\boldsymbol{\Delta }}{\rm{L}}$ is the optical path difference between the two arms of the MZI.

Based on the fact that the linear phase shift is frequency-dependent, the transmission spectrum of the interferometer relies on the wavelength of the light and the FSR is mainly decided by ${\boldsymbol{\Delta }}{\rm{L}}$. When the optical path different increases from ${\boldsymbol{\Delta }}{\rm{L}}$ to ${\boldsymbol{\Delta }}{\rm{L}}+{\boldsymbol{\Delta }}{{\rm{L}}}_{1}$, the wavelength shift can be calculated and expressed as:

2 $${\boldsymbol{\Delta }}\lambda =\frac{{\lambda }_{0}}{{\boldsymbol{\Delta }}{\rm{L}}}{\boldsymbol{\Delta }}{{\rm{L}}}_{1}$$

where ${\lambda }_{0}$ is the wavelength of the operation peak and ${\boldsymbol{\Delta }}{{\rm{L}}}_{1}$ is the variation of the optical path difference.

**Figure 2:**
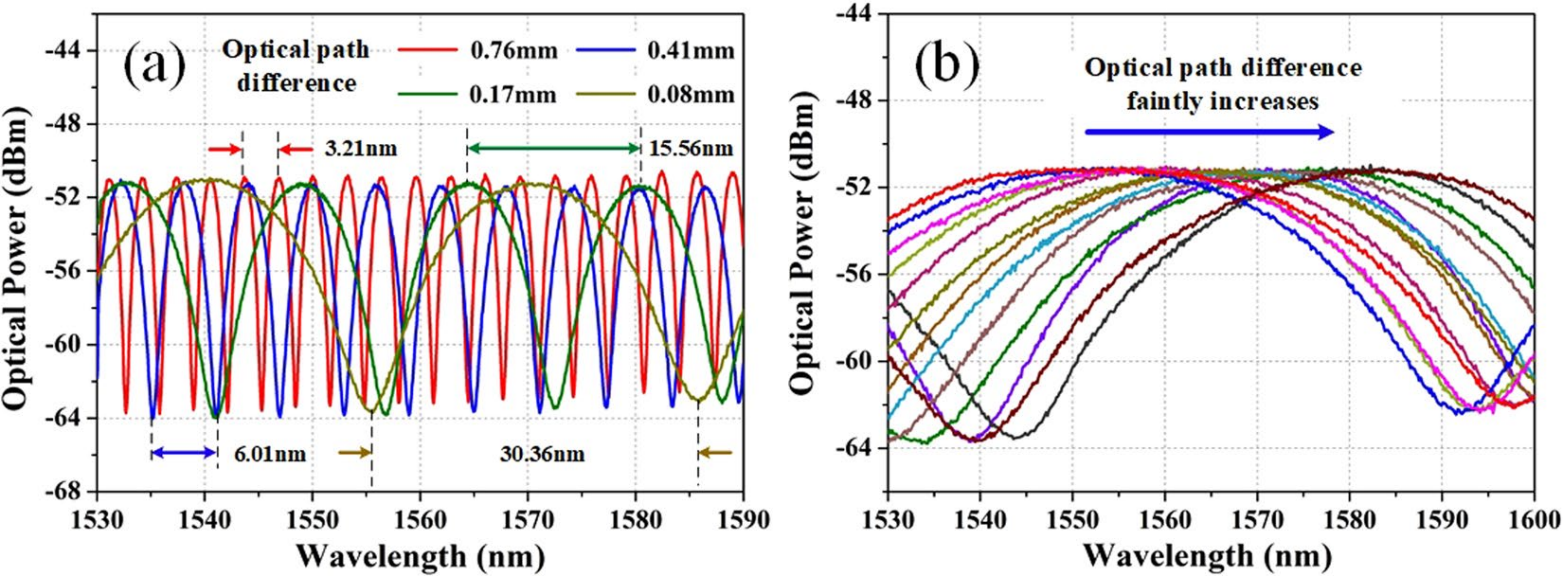
Optical filtering characteristics of the MZI. (**a**) MZI transmission spectra with optical path difference at 0.76 mm (red line), 0.41 mm (blue line), 0.17 mm (green line), and 0.08 mm (brown line) whose corresponding FSRs are 3.21 nm, 6.01 nm, 15.56 nm and 30.36 nm, respectively; (**b**) wavelength tunes while the optical path difference faintly increases.

By injecting light with the power of 12 dBm from a supercontinuum source, optical power of the output light is measured as 10 dBm which means an optical propagation loss of 2 dB through the MZI. Then, the transmission spectra are recorded with the optical path differences of 0.76 mm (red line), 0.41 mm (blue line), 0.17 mm (green line) and 0.08 mm (brown line) as shown in Fig. 2(a). And the corresponding FSRs are measured as 3.21 nm, 6.01 nm, 15.56 nm and 30.36 nm that agree with Eq. (1). It means that the FSR can be accurately controlled by adjusting the OVDL and contribute to managing the pulse bandwidths in the fiber laser. Subsequently, we set the FSR at 70 nm and tune OVDL faintly. A continuous wavelength red-shift is observed and recorded as depicted in Fig. 2(b). Thus, it is evident that the transmission peak can be flexibly tuned over a large range by finely adjusting the OVDL; while the FSR basically remains unchanged. Consequently, a wavelength-tunable fiber laser can be constructed based on the MZI.

**Figure 3:**
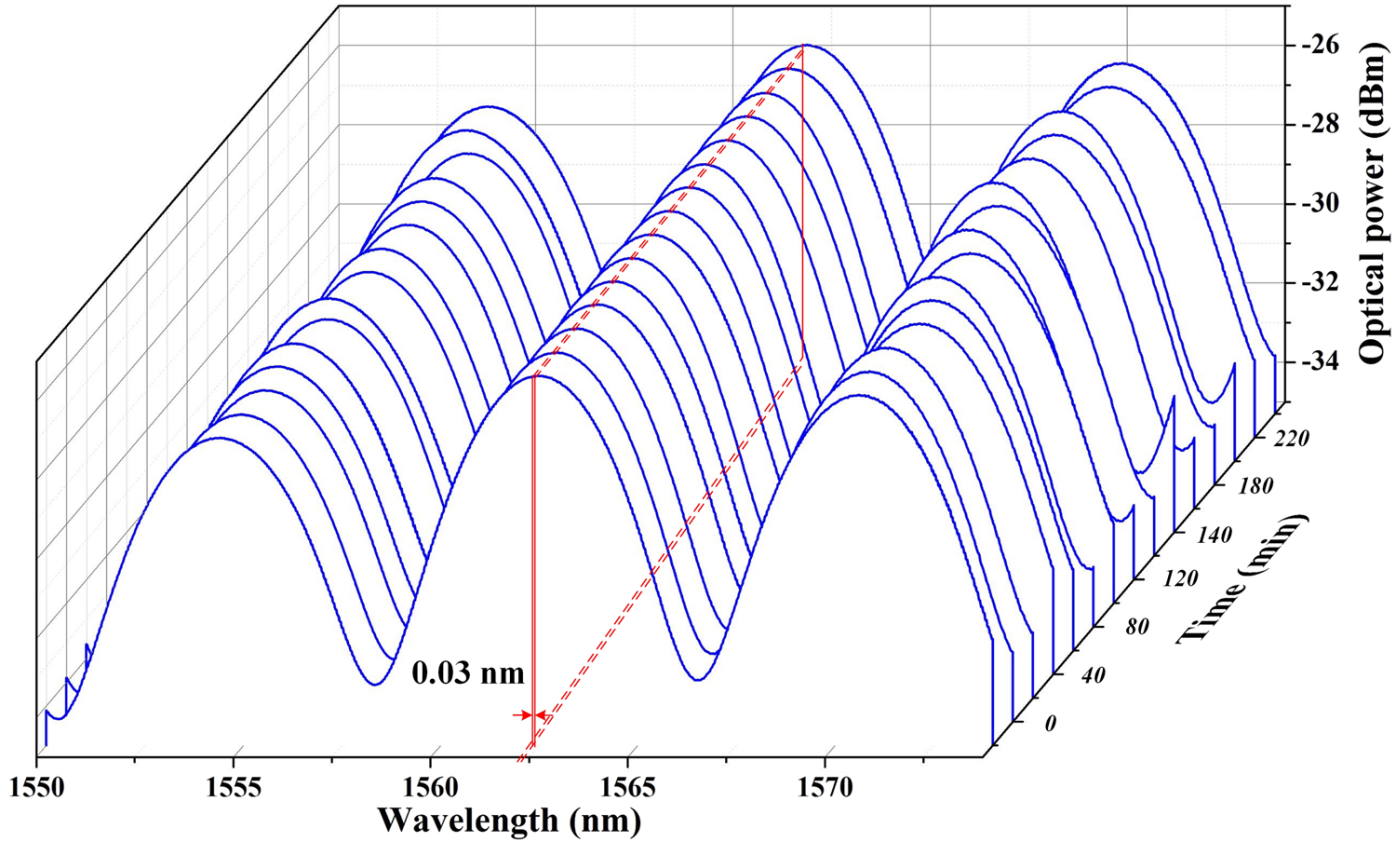
The measured results for the stability of the MZI, where the transmission output spectrum is monitored every 20 mins in 4 hours.

To perform a stability analysis on the MZI, the transmission spectrum is recorded at an interval of 20 minutes for 4 hours, as displayed in Fig. 3. It can be seen that the spectra maintain relatively stable and no significant variations in the optical power. Though slightly fluctuation is observed in FSR, the peak at 1562 nm only has a central wavelength variation of 0.03 nm, varying from 1562.24 nm to 1562.27 nm. It should be noted that the location of the peak wavelength can be controlled by finely adjusting the OVDL. The additional slight fluctuations of 0.03 nm may be caused by slight vibration of the ambient environment. Hence, the stability of the proposed fiber laser can be improved by optimizing the MZI configuration.

### Laser setup

**Figure 4:**
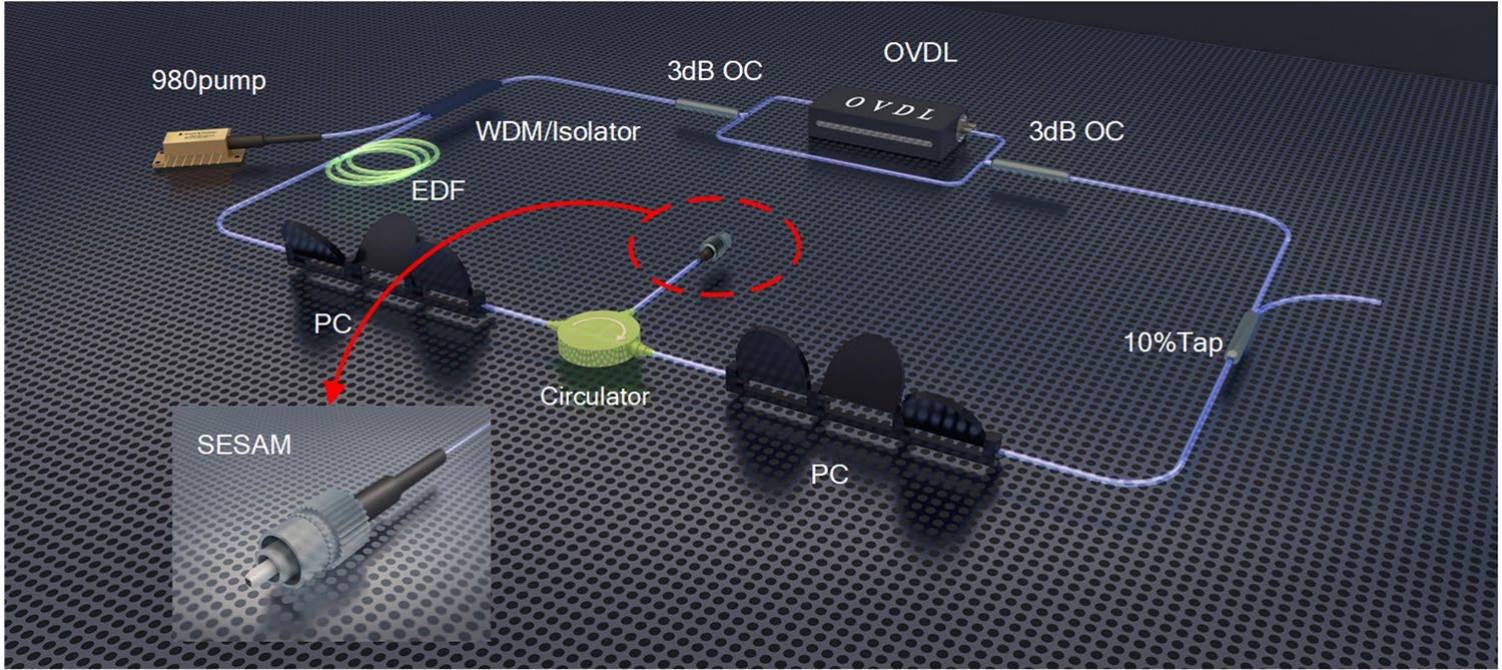
Schematic diagram of the experimental setup. 10%Tap, 10:90 output coupler; PC, polarization controller; SESAM, semiconductor saturable absorber mirror; EDF, Erbium-doped fiber.

The fiber laser is schematically illustrated in Fig. 4. The mode-locking is achieved based on a fiber-pigtailed semiconductor saturable absorber mirror (SESAM, BATOP, saturable absorption of 16%, modulation depth of 9%, and recovery time of 2 ps). By using a three-port circulator, the SESAM is incorporated into the cavity. A wavelength-division multiplexer/Isolator (WDM/Isolator) hybrid module is utilized to simplify the laser configuration. A 1.5 m erbium-doped-fiber (EDF) (Fibercore I25) is chosen as the gain medium and it is pumped by a 980 nm laser diode (LD) through the (WDM/Isolator) hybrid module. The intra-cavity polarization state is finely tuned by the two fiber-based polarization controllers (PCs). A 90:10 fiber optical-coupler (OC) is used at the output port. Especially, an MZI, consisting of two 3 dB OCs and an optical variable delay line (OVDL), is applied between the output OC and the WDM/Isolator hybrid module, which acts as an intra-cavity filter to realize the wavelength-tuning of the fiber laser. The EDF used in our experiment has a group-velocity dispersion (GVD) of about −18 (ps/nm)/km, all optical devices are connected by single-mode fibers (SMFs) with GVD of about +17 (ps/nm)/km, and the total length of the cavity is around 30.5 m. Consequently, the net dispersion of the cavity is about −0.605 ps^2^.

### Multipulse states

**Figure 5:**
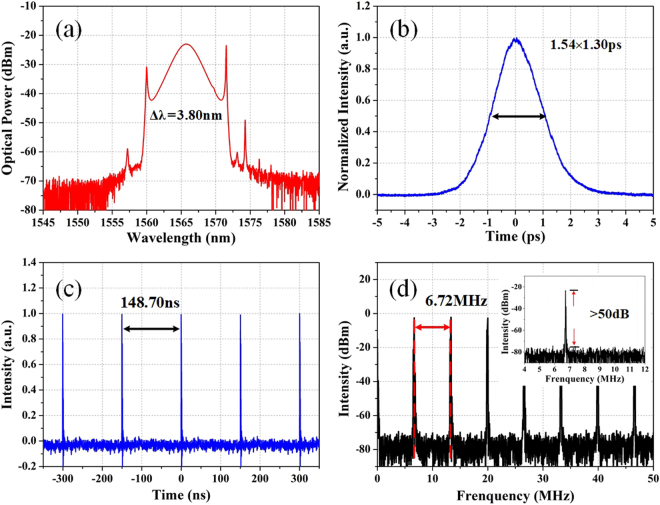
Conventional solitons. (**a**) Optical spectrum; (**b**) autocorrelation trace; (**c**) oscilloscope trace and (**d**) RF spectrum at the fundamental mode-locking operation (inset is detailed RF spectrum).

To start with, the optical path difference between the two arms of the MZI is artificially fixed. With suitable setting, mode-locking can self-start along with the pump power increasing over the mode-locking threshold of 23 mW and appropriately adjusting the paddles of the PCs. When the pump power is adjusted to 132 mW, the optical spectrum depicted in Fig. 5(a) exhibits a typical soliton spectral shape with a 3-dB bandwidth of 3.80 nm. As shown in Fig. 5(b), the autocorrelation trace indicates a pulse width of 1.30 ps if a sech^2^ pulse shape is assumed. Thus, the time-bandwidth product (TBP) is around 0.61 which implies slightly chirped the pulse is. Besides, the oscilloscope trace and radio frequency (RF) spectrum at single-pulse work state are presented in Fig. 5(c) and (d) separately. The pulse interval is around 148.7 ns, exactly agreeing with the repetition rate of 6.72 MHz. Furthermore, the average output power at fundamental mode-locking operation is about 68 μW, which implies the single-pulse energy of 9.97 pJ.

**Figure 6:**
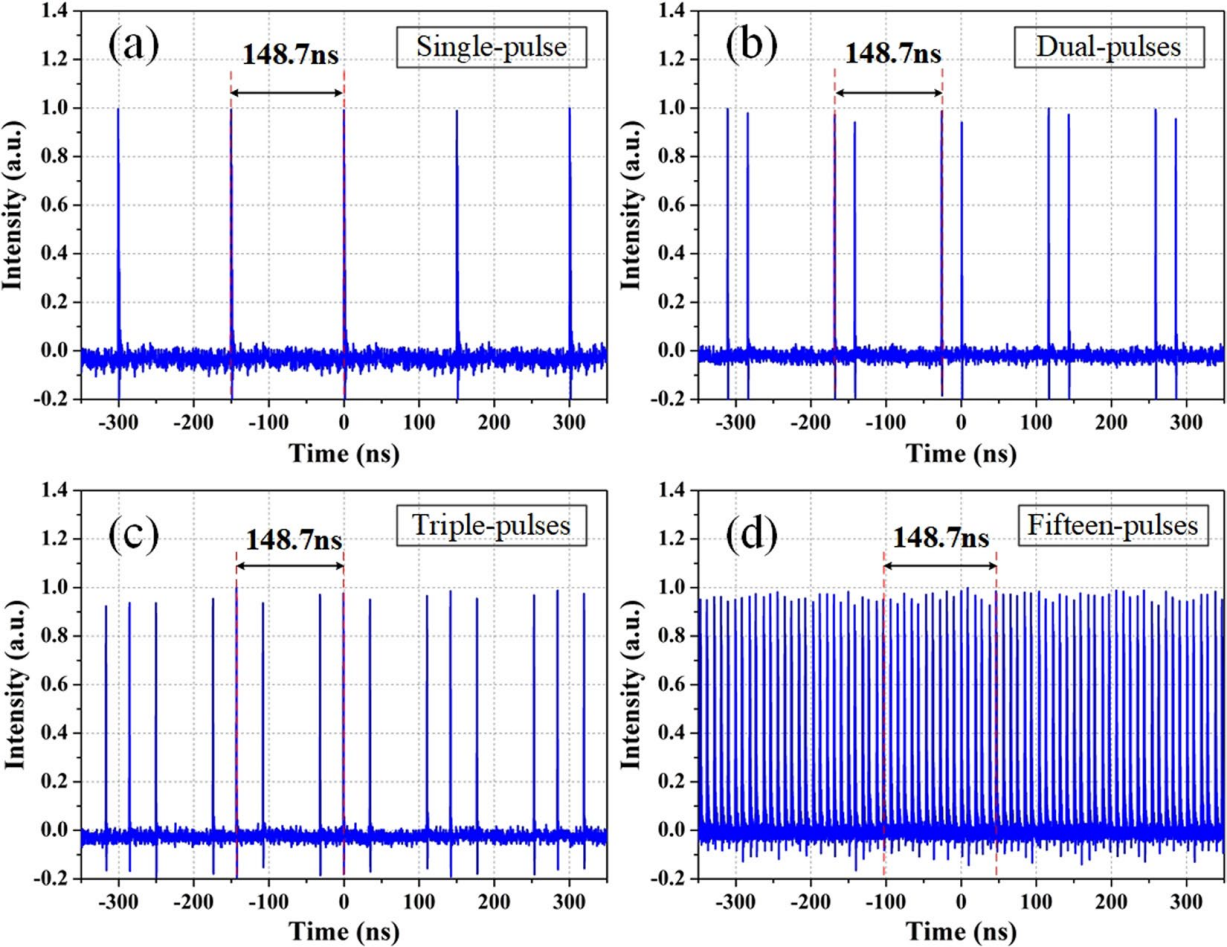
Oscilloscope traces of the multi-pulses states. (**a**) Single-pulse (**b**) dual-pulse (**c**) triple-pulse and (**d**) fifteen-pulse states in higher pump.

With the pump power increasing, the fundamental operation regime is broken into the multi-pulse states under the limitation of the soliton energy quantization effect. As a result, the operation states of single, dual and triple pulses are experimentally recorded as shown in Fig. 6(a–c). When the pump power continues increasing, the fifteen pulses appear as shown in Fig. 6(d). And these particle-like independent solitons provide the possibility to generate the bound states of solitons.

### Wavelength tuning operation

**Figure 7:**
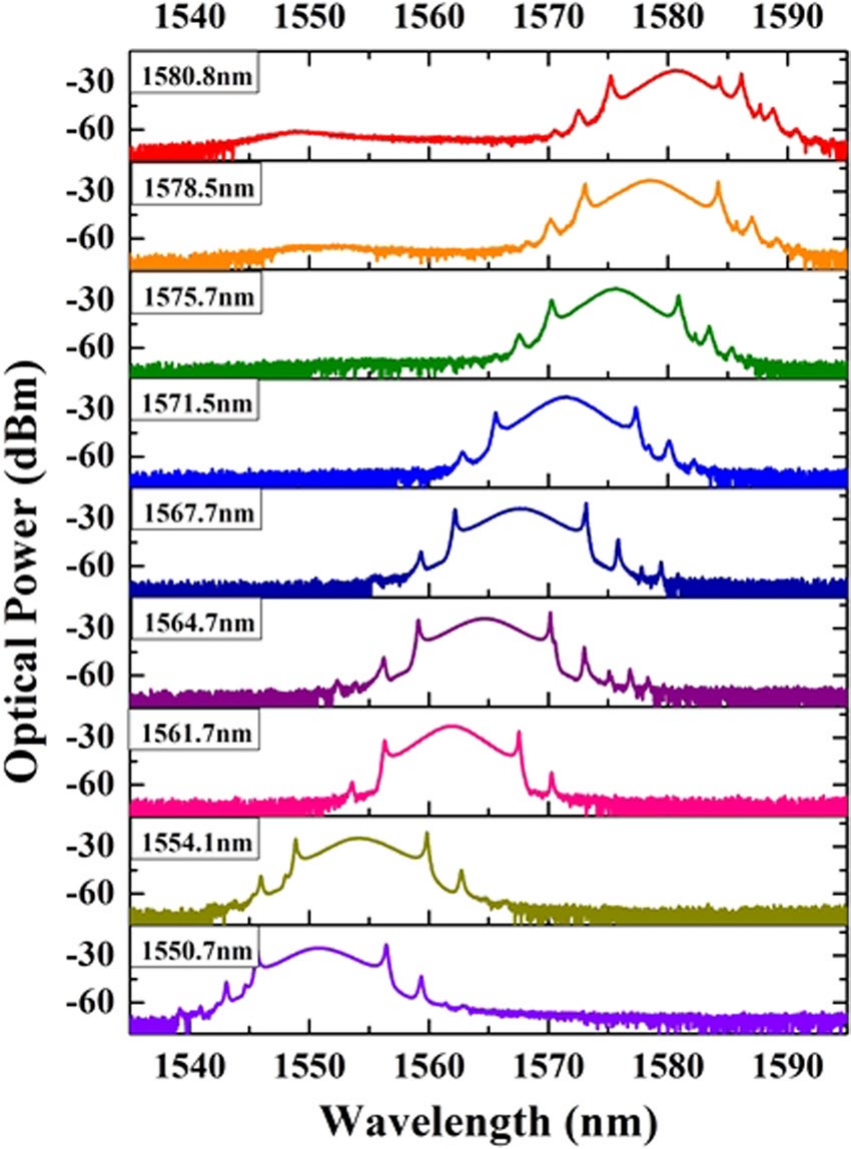
Wavelength tuning operation of the fiber laser.

By finely tuning the OVDL, optical path difference between the two arms is controllably changed which leads to the wavelength shift of the intra-cavity filter as demonstrated in Eq. (2) and Fig. 2(b). In the experiment, the FSR of the MZI transmission spectra was set to 150 nm firstly and the mode-locking is achieved by properly adjusting the paddles of the PCs. With finely tuning the OVDL, Fig. 7 specifically illustrates the tunable optical spectra of the solitons at 9 designated wavelengths of 1550.7 nm, 1554.1 nm, 1561.7 nm, 1564.7 nm, 1567.7 nm, 1571.5 nm, 1575.7 nm, 1578.5 nm and 1580.8 nm which varies from C band to L band covering more than 30 nm. It is obvious that spectral shape of the solitons remains almost the same, and no significant differences are observed for the pulse trains. Thus it can be seen that continuous wavelength-tunability of the fiber laser is experimentally obtained through exploiting a MZI as the intra-cavity filter. However, it is found that the spectra will distort when the center wavelength is over 1580.8 nm or below 1550.7 nm. The limitation of 30-nm tunable range is ascribed to the excitation spectral width of the EDF.

### Pulse bandwidth control

**Figure 8:**
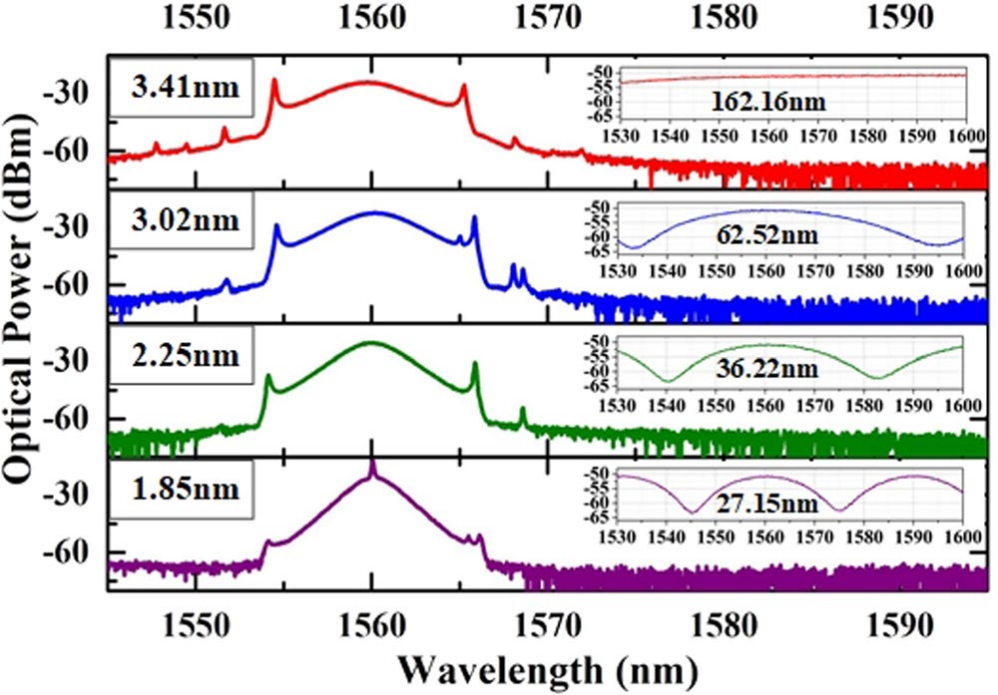
Experimental results of spectrum bandwidth variation (insets are the transmission spectra of the MZI).

To further study the function of MZI in the fiber laser, we conduct the extended experiment in which the OVDL is adjusted roughly. As we known, the optical path difference can be obviously changed with the variation of the inline OVDL. As depicted in Fig. 8, the 3-dB bandwidth of the laser spectra decreases from 3.41 nm to 1.85 nm along with the transmission spectra of MZI narrowing down from 162.16 nm to 27.15 nm accordingly, which means that the spectral bandwidth is controllable. The spectral bandwidth is thought to be influenced by two reasons. Mode-locked mechanism limits the 3-dB-bandwidth when the FSR of MZI is broad enough. On the contrary, the filtering effect will play a crucial role instead of the mode-locked mechanism when the FSR is narrow.

### Loosely/tightly bound states

**Figure 9:**
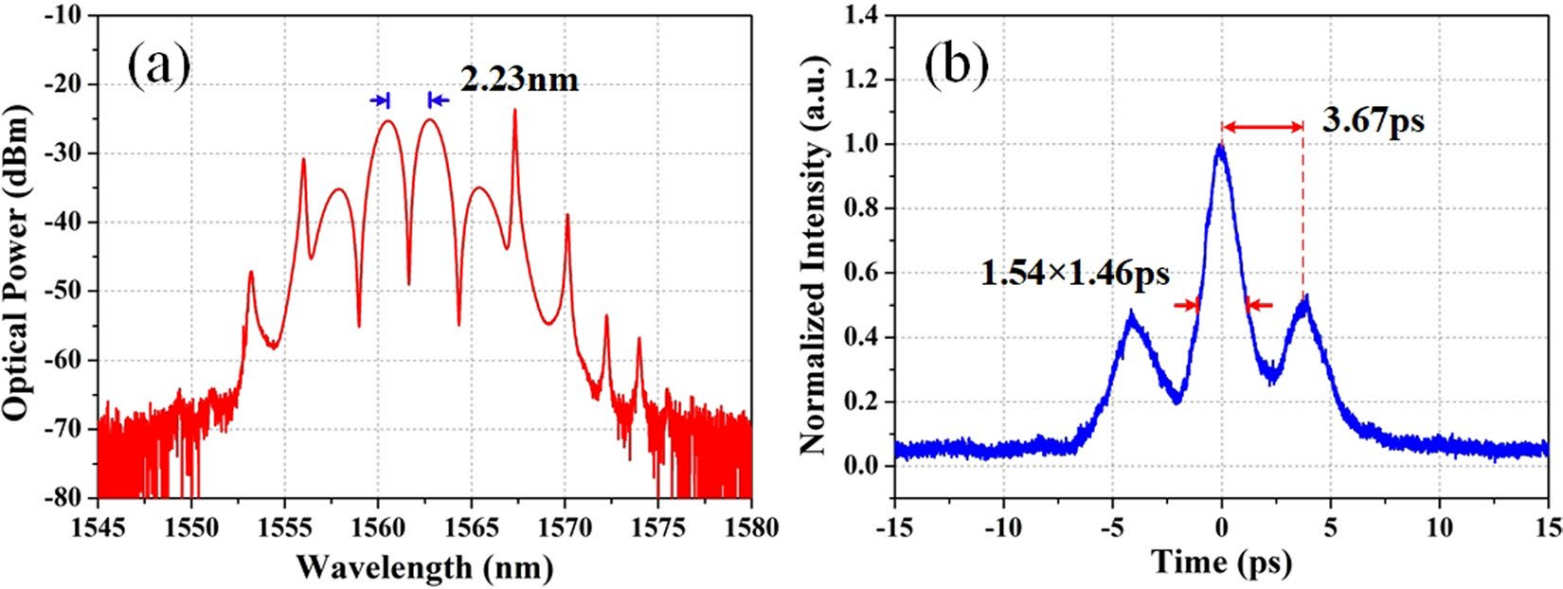
Soliton bound state. (**a**) Optical spectrum of the tightly bound state; (**b**) corresponding autocorrelation trace of the bound state.

Through increasing the pump power to ~110 mW, setting the FSR of the MZI to 500 nm and properly adjusting the paddles of the PCs, the separate solitons could be bound together, thus forming the soliton bound states on account of the balance of repulsive and attractive forces caused by nonlinearity and dispersion^25^. As depicted in Fig. 9(a), the corresponding spectrum centered at 1562 nm exhibits the typical characteristic of bound solitons, showing obvious spectral modulation. Meanwhile, the 2.23-nm FSR reveals that the two solitons are closely spaced. Further, the soliton molecules can be analyzed based on the corresponding autocorrelation trace as depicted in Fig. 9(b). The pulse width is 1.46 ps with assumed sech^2^ pulse shape, and the solitons separation is about 3.67 ps, which is in good agreement with the spectral fringe. The solitons separation is estimated to be only 2.5 times of the pulse width, which can be considered as a tightly bound state.

**Figure 10:**
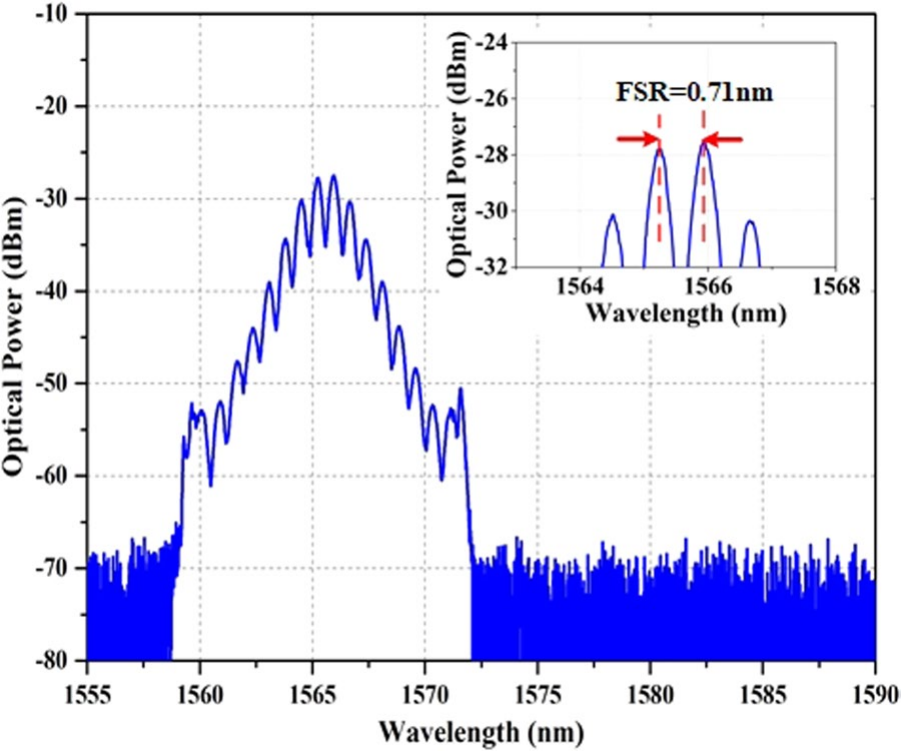
Optical spectrum of loosely bound state (inset is the detailed information).

Additionally, under pump power of ~200 mW and appropriate PC orientation, another soliton bound state, namely the loosely bound state, is also experimentally observed in this fiber laser with the fine adjustment of the OVDL. As illustrated in Fig. 10, the spectrum possesses a modulation period of 0.71 nm which implies a soliton separation of 11.65 ps. It is around 8.3 times of the pulse width which is supposed to be 1.4 ps. Although we do not verify the pulses separation directly by measuring autocorrelation trace for the low optical power of output light, the following numerical simulations provide sufficient information for our judgment.

## Discussions

**Figure 11:**
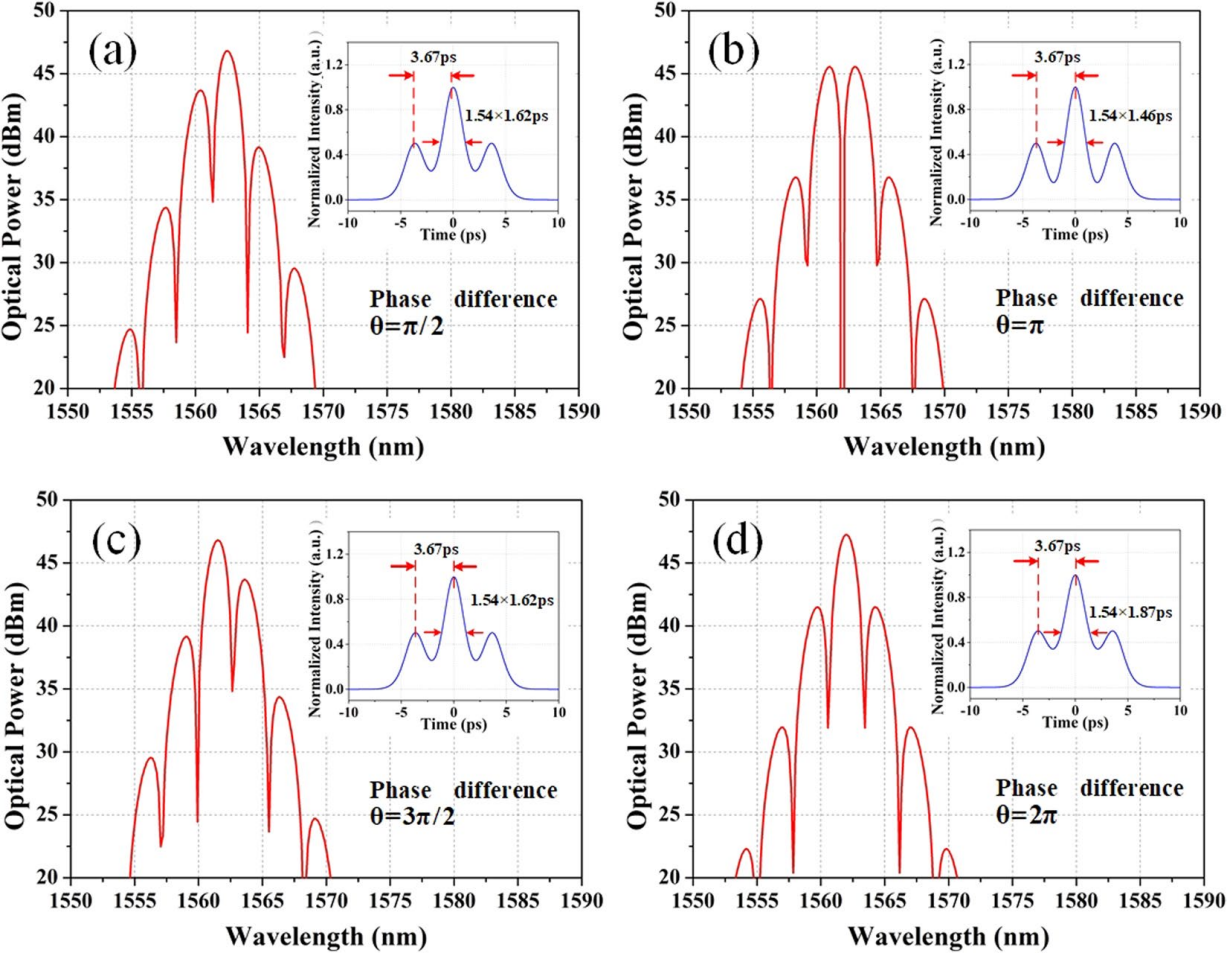
Numerical simulations of bound states with different phase-difference. (**a**) π/2, (**b**) π, (**c**) 3π/2, (**d**) 2π (insets are the corresponding autocorrelation traces).

Through controlling the pump power and properly manipulating the paddles of the PCs, tightly and loosely bound states have been experimentally observed. The separation and width of solitons are also acquired from the autocorrelation trace. However, the phase difference of the solitons which also has a significant impact in the formation of soliton bound states is still unknown. Thus, numerical simulations of the laser operation are carried out as follow. Firstly, based on the experimental results in Fig. 8, the separation and pulse width of solitons are assumed to be 3.67 ps and 1.46 ps. The spectra with phase differences of π/2, π, 3π/2 and 2π are numerically recorded as depicted in Fig. 11(a–d). The soliton molecules with phase difference of π/2 and 3π/2 have asymmetrical spectra. On the contrary, the soliton molecules with phase difference of π and 2π have symmetrical spectra with respect to the center wavelength. In detail, the former has a dip in the center, while the latter has a peak in the center. Thus, it is verified that the spectral symmetry is decided by the phase relationship of the pulses. Compared with our experimental results, the tightly bound state is confirmed to be formed by two solitons with the phase difference of π.

**Figure 12:**
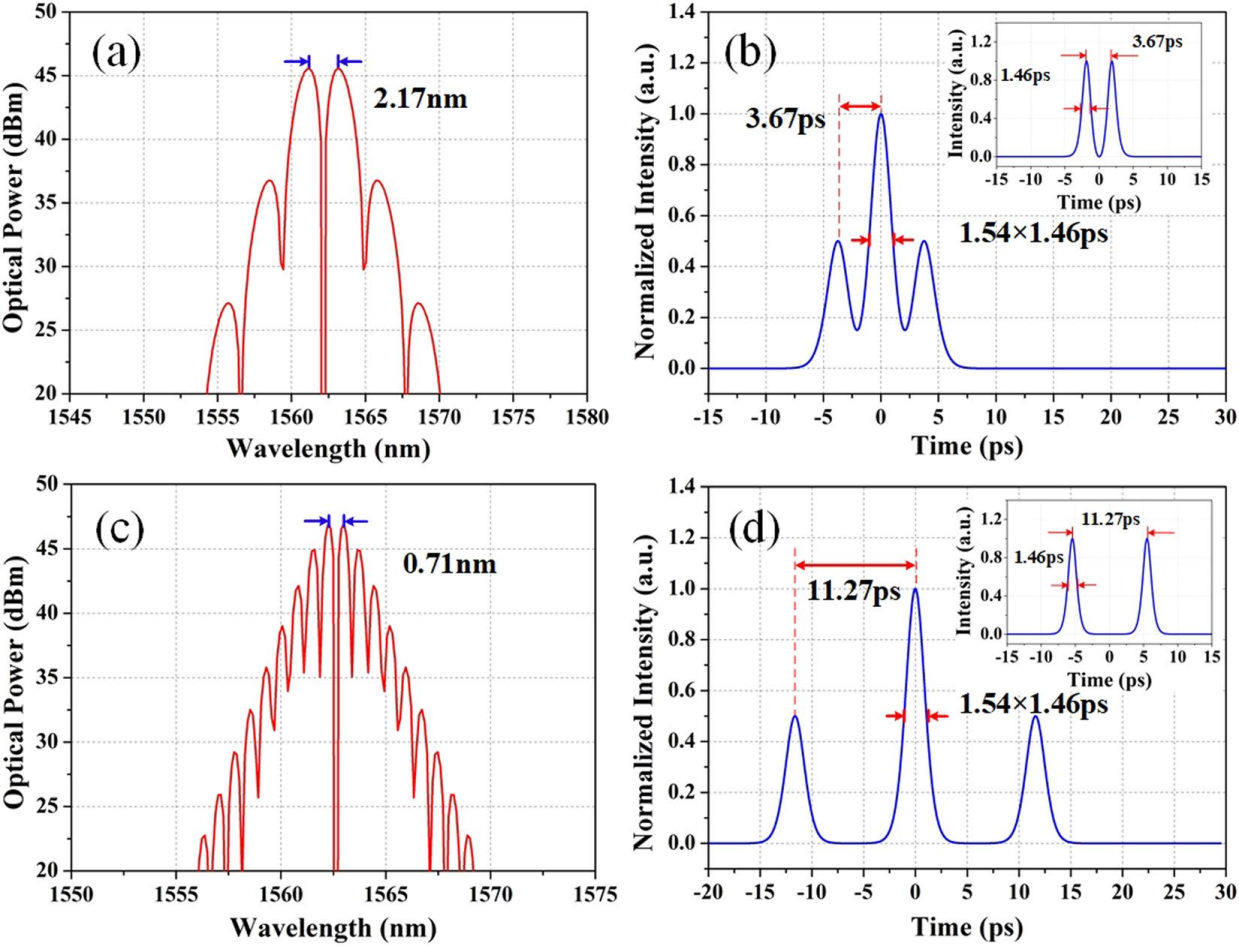
Numerical simulations of bound states with different pulses separations. (**a**) Spectrum and (**b**) autocorrelation trace of the tightly bound state (inset is the pulses state); (**c**) spectrum and (**d**) autocorrelation trace of the loosely bound state (inset is the pulses state).

Apart from phase difference, the separation of solitons can also influence the spectrum of lasers. Thus, the numerical simulation with different separations of solitons is implemented. As depicted in Fig. 12(a,b), the spectrum shows a modulation period of 2.17 nm with the pulses separation of 3.67 ps which is well fit with the tightly bound state as shown in Fig. 9. And when the pulse separation extends to 11.27 ps which is 7.72 times of pulse width, the spectrum exhibits a denser modulation of 0.71 nm that is in agreement with the experimental results in Fig. 10. Hence, it is confirmed that the modulation periods of spectra depend on the separations of the pulses and the loosely bound state proves to be achieved in the same fiber laser and bound with two sech^2^-shape solitons with the separation of 11.27 ps and pulse-width of 1.46 ps.

In conclusion, we investigate wavelength tuning and bound states in the same fiber laser. Through finely adjusting the MZI intra-cavity filter, the optical spectra of the solitons can be tuned from 1550.7 nm to 1580.8 nm while the spectral bandwidth varying from 1.85 nm to 3.41 nm is controlled by changing the FSR of MZI in a large scale. Additionally, with modest polarization manipulation, both tightly and loosely bound states are experimentally observed, which can also be validated by the numerical simulations. The MZI-based fiber laser can serve as an ideal playground for exploring the interesting behaviors and dynamics of DSs and can be applied to telecommunication systems whose tuning feature can be applicable to WDM and the various soliton bound states could contribute to the high-level modulation format.

## Methods

An optical spectrum analyzer (OSA, Yokogawa AQ6370D), a 10 GHz real-time oscilloscope (OSC, Tektronix CSA7404B), a 40 GHz electrical spectrum analyzer (ESA, Agilent E4447A), a 1.6 GHz photodetector (PD, Thorlabs PDB480C-AC), and a second harmonic generation (SHG) autocorrelator (FEMTOCHROME FR-103XL, resolution <5 fs) are used to measure the laser output performances.
